# Saw-Tooth Cardiomyopathy: the Evidence in the First Decade

**DOI:** 10.31083/j.rcm2304138

**Published:** 2022-04-11

**Authors:** Zhiyu Liu, Yingying Zheng, Jinying Zhang

**Affiliations:** ^1^Cardiology Department, the First Affiliated Hospital of Zhengzhou University, 450052 Zhengzhou, Henan, China; ^2^Key Laboratory of Cardiac Injury and Repair of Henan Province, 450052 Zhengzhou, Henan, China; ^3^Henan Province Clinical Research Center for Cardiovascular Diseases, 450052 Zhengzhou, Henan, China

**Keywords:** saw-tooth cardiomyopathy, cardiac magnetic resonance, left ventricular noncompaction

## Abstract

Saw-tooth cardiomyopathy (STC), a rare form of left ventricular cardiomyopathy characterized by saw-tooth like myocardial projections extending from the lateral walls towards the ventricular cavity, is a newly discovered cardiomyopathy first described in 2009. Detailed cardiac magnetic resonance can demonstrate multiple dense myocardial protrusions originating from the inferior wall, interventricular septum and lateral ventricular walls, which differ from typical left ventricular noncompaction. STC case reports are increasing since the first discovery. A total of ten cases have been reported. This review focuses on the clinical presentation and imaging features of this disease and analyzes the latest evidence regarding STC. Furthermore, we summarize the clinical evidence from the current decade, which may enhance detection and diagnosis of this disease in the future.

## 1. Introduction

**Fig. 1.**
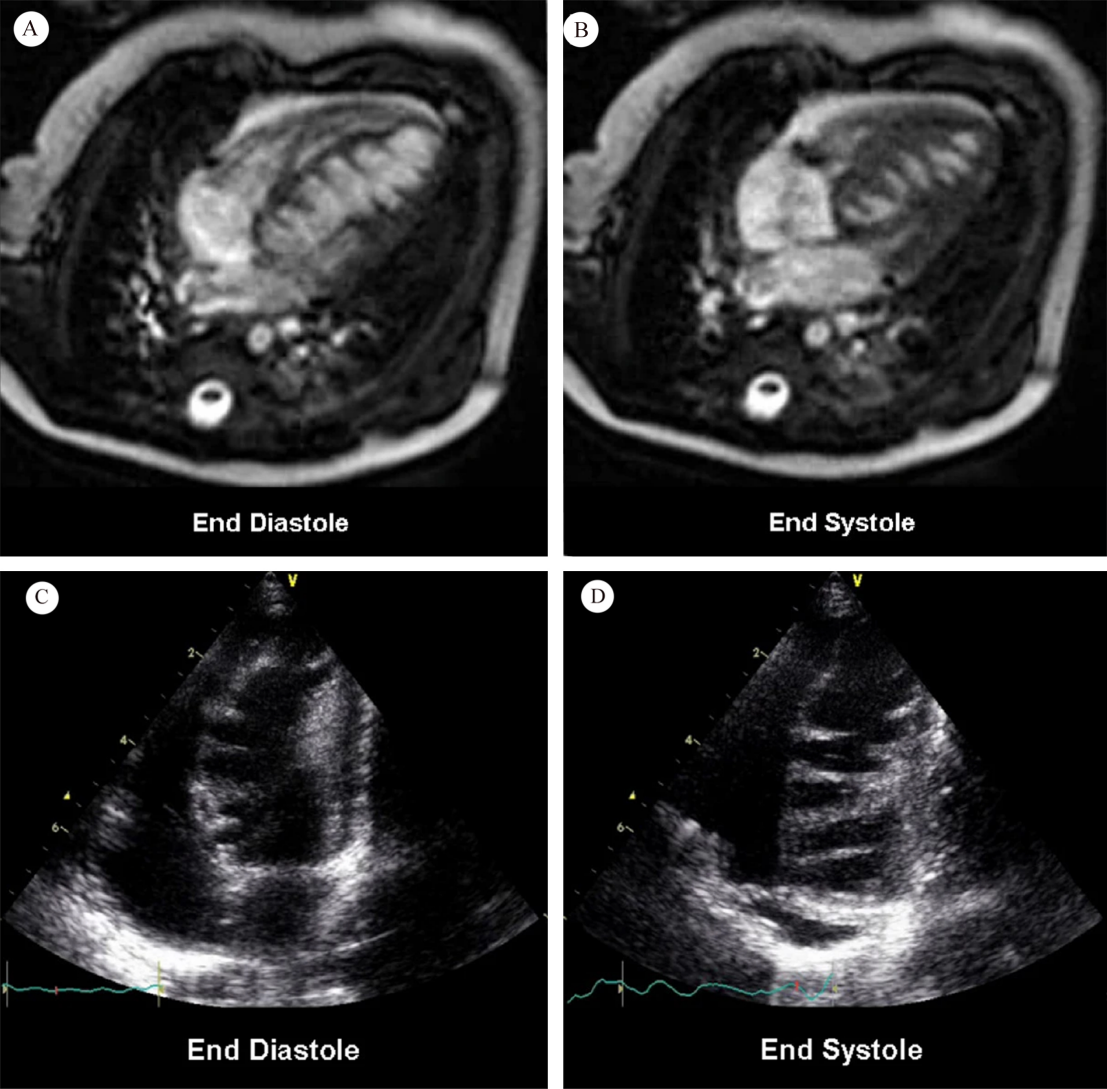
**Cardiac magnetic resonance (A and B); transthoracic echocardiography (C and D)**. Fig. 1A,B show numerous saw-tooth like muscular projections originating from the interventricular septum, some of them being tethered to the inferior LV wall. Fig. 1C,D show numerous saw-tooth like projections originating from the inferior interventricular septum to lateral LV wall (Fig. 1 is cited from Davlouros PA, *et al*. [2]).

**Fig. 2.**
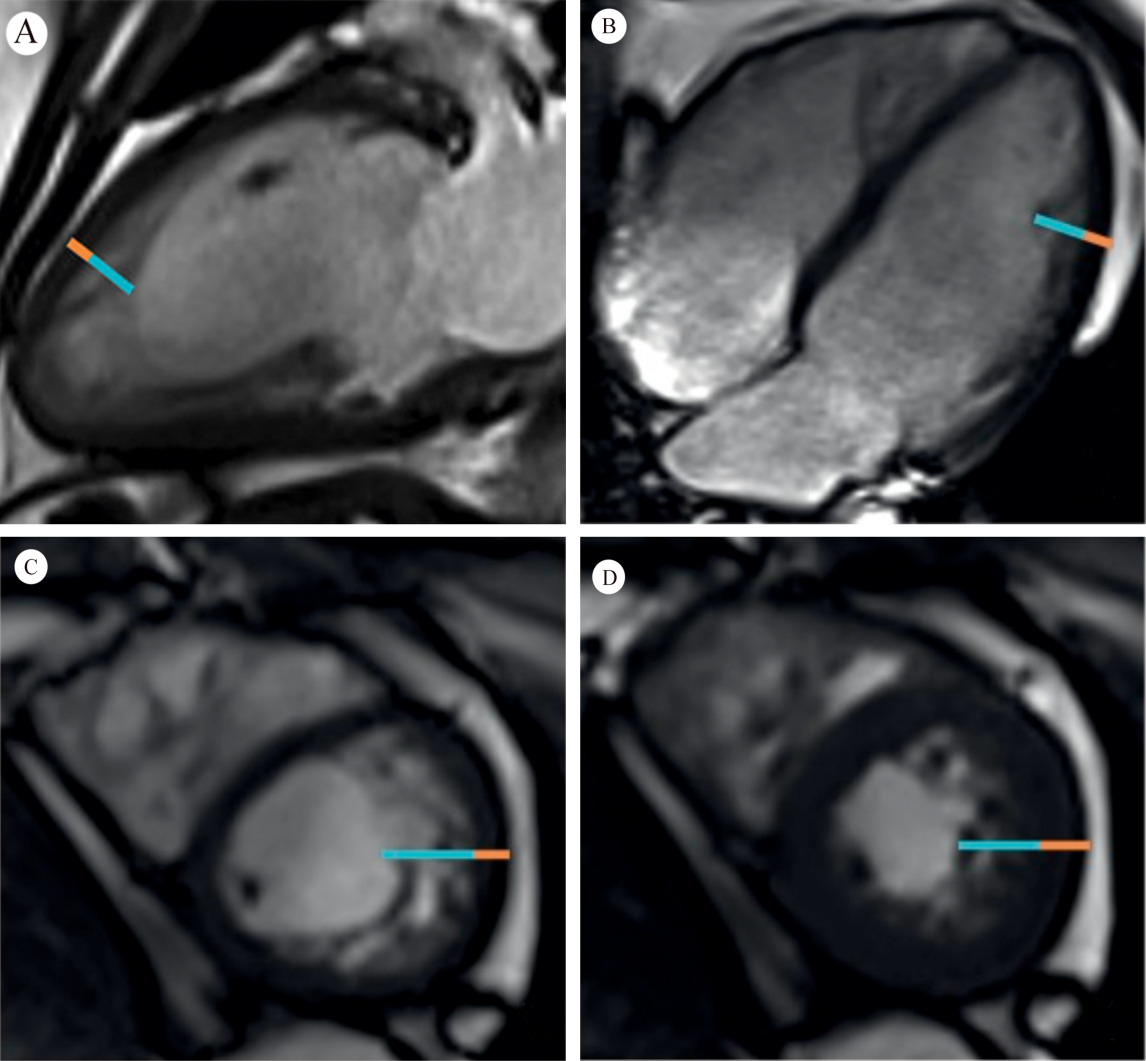
**Orange line = compacted myocardium; Blue line = noncompacted myocardium**. Fig. 2A,B demonstrate long axis noncompaction ratio measurement, with a maximum ratio of 3.4. Fig. 2C,D demonstrate a short axis noncompaction ratio at diastole of 3.6, and a systole of 2.2, respectively (Fig. 2 is cited from Weir-McCall JR, *et al*. [3]).

Saw-tooth cardiomyopathy (STC), first discovered in 2009 [1, 2], is a rare form of left ventricular cardiomyopathy, mainly affecting the apex and middle segment of the left ventricular wall and inferior wall. It may be evident in transthoracic echocardiography on admission. In most reported cases cardiac magnetic resonance (CMR) can provide a clearer view of the internal structure of the heart, with multiple dense myocardial protrusions similar to saw-tooth like crypts. These differ from left ventricular noncompaction (LVNC), which is characterized by noncompact myocardium in the layer of finely trabeculated myocardium adjacent to a layer of compacted myocardium [3]. The clinical course of STC ranges from asymptomatic to acute heart failure, and it can also be associated with a variety of cardiac complications. Typical images for STC are shown in Fig. 1 (Ref. [2]), and for LVNC in Fig. 2 (Ref. [3]) for comparison.

STC case reports have been increasing in the last decade since the first discovery. Case studies have focused on the imaging manifestations of the disease and abnormal myocardial structure. Echocardiogram and CMR scanning are increasingly used in STC examinations, while long-term monitoring and outpatient treatment have important roles in STC management. However, to date there is no systematic review of STC features. This article reviews the latest developments in this area, presenting the imaging manifestations of STC and analyzing the latest evidence. Additionally, this review summarizes current clinical features in order to develop diagnostic strategies for patients with STC.

## 2. History of Saw-Tooth Cardiomyopathy Discovery

In March 2009, Isma Rafiq *et al*. [1] reported a case entitled “A Previously Undescribed Variant of Isolated Left Ventricular Noncompaction” in the *Journal of the American College of Cardiology*. In this case of a 37-year-old male patient with chest pain, left bundle branch block was found on an electrocardiogram, and an echocardiogram demonstrated deep trabeculation of the left ventricular septum. Most importantly, CMR revealed significant myocardial manifestations different from classic LVNC: abnormal basal hypertrophy of the ventricular septum towards the apex of the heart with multiple myocardial crypts in the extension of the basal part, and multiple band-like myocardial tissues extending from the inferior septum to the ventricle.

In December 2009, Periklis A Davlouros *et al*. [2] published a case report titled “Saw tooth cardiomyopathy” (Fig. 1). A heart murmur and abnormal brain natriuretic peptide level of 352 pg/mL (normal <32.7 pg/mL) were found in a 2-month-old male infant. Transthoracic electrocardiogram showed mild left ventricular systolic insufficiency, hypokinesia of the ventricular septum, ventricular aneurysm on the apex, patent foramen ovale with left-to-right shunt and, most significantly, numerous echo dense saw-tooth like protrusions arising from the inferior septum and the left ventricular lateral wall extending into the cavity. CMR confirmed a large number of cross-bridging muscular protrusions from the inferior wall, inferior interventricular septum, and left ventricular sidewall.

In 2012, Anoop C. Parameswaran *et al*. [4] described in detail a CMR result of an 80-year-old man with heart failure (LVEF 38%), titled “Tiger Heart”. They described that the prominent and deep trabeculae originate from the interventricular septum, with associated thinning and hypokinesia of the anterior, apical, and distal inferior walls, resembling tiger stripes, and that late gadolinium-enhancement imaging showed no myocardial hyperenhancement.

In 2020, Pablo Garcia-Pavia [5] published an editorial comment in *JACC: Case Reports* titled “Saw-Tooth Cardiomyopathy”, emphasizing the morphological traits of compacted myocardium with evident cross-bridging muscular projections on CMR and echocardiogram, and recommending that such new findings in patients with “myocardial cross-bridging protrusions” be collectively referred to as “saw-tooth myocardium”. We summarize all reported saw-tooth cardiomyopathy and Tiger heart cases of which we are aware in Table 1 (Ref. [1, 2, 4, 6, 7, 8, 9, 10, 11, 12]).

**Table 1.** **Saw-tooth cardiomyopathy case reports from year 2009 to 2021**.

Reporter	Time	Demographic	CC/HF parameter	Associated features	Treatment plan
Rafiq I [1]	Year 2009	37-year-old (male)	musculoskeletal chest pain	LBBB	Follow-up 6 months
Davlouros PA [2]	Year 2009	2-month-old (male)	BNP 352 pg/mL	LVA, PFO	Digitalis, carvedilol, furosemide and an ACEi; Follow-up 6 months
Parameswaran AC [4]	Year 2012	80-year-old (male)	LVEF 38%	-	-
Cardoso BDP [6]	Year 2017	21-year-old (male)	Congestive heart failure	-	-
Chenaghlou M [7]	Year 2020	32-year-old (male)	LVEF 35%	CMB	Follow-up every 3 months
Bailly MT [8]	Year 2020	75-year-old (male)	LVEF 57%	TIA, HTN, HL	Aspirin, statins, antihypertensive drugs
Halioui M [9]	Year 2020	16-year-old (female)	LVEF 68%	MVP, mMR	Expected surgical protocol
Proukhnitzky J [10]	Year 2020	33-year-old (male)	LVEF 55%	LPFB	Aspirin and PPI; Follow-up 25 months
Wegner FK [11]	Year 2020	30-year-old (male)	LVEF 48%	LBBB	Follow-up 52 months
García-Ropero Á [12]	Year 2021	34-year-old (female)	Congestive heart failure, pneumonedema	HTN, AS, LBBB	Emergency arterial thrombolysis (left middle cerebral artery), oral anticoagulants, and CRT device implantation

CC, chief compliant; HF, heart failure; LBBB, left bundle branch block; BNP, brain natriuretic peptide; ACEi, angiotensin converting enzyme inhibitor; LVEF, left ventricular ejection fraction; LAVA, left apical ventricular aneurysm; PFO, patent foramen ovale; CMB, coronary myocardial bridge; TIA, transient ischemic attack; HTN, hypertension; HL, hyperlipidemia; MVP, mitral valve prolapse; mMR, moderate mitral regurgitation; LPFB, Left posterior branch block; PPI, proton pump inhibitor; AS, acute stroke; CRT, cardiac resynchronization therapy; -, the original literature has not reported.

## 3. Current Clinical Features of Saw-Tooth Cardiomyopathy

The precise pathophysiological mechanism of STC is incompletely understood, so findings of it can be applied only to clinical diagnosis at present. The diagnosis of STC is often challenging because its clinical phenotype may closely resemble LVNC regarding symptoms, echocardiogram and ECG abnormalities. While a widely established non-invasive tool allowing a rapid and reliable diagnosis of STC is currently lacking, CMR with late gadolinium-enhancement imaging is considered the standard diagnostic tool to exclude or confirm STC:

Proposed Criteria for the Clinical Diagnosis of STC:

(1) Saw-tooth or band-like projections originating from the interventricular septum to mid lateral segments, with apparently compact myocardium;

(2) Mild impaired left ventricular contraction function is usually clinically diagnosed with heart failure;

(3) Electrocardiogram is generally normal, or with left bundle branch block;

(4) STC can be accompanied by congenital heart disease, including left apical ventricular aneurysm, patent foramen ovale, coronary myocardial bridge, and mitral valve prolapse.

## 4. Imaging Evaluation of Saw-Tooth Cardiomyopathy

When STC patients were inadvertently discovered, the echocardiogram imaging of saw-tooth like projections in the ventricle usually did not match the classic LVNC signs, so clinicians commonly chose to use cardiac magnetic resonance (LVNC Petersen standard [13]) for an accurate analysis of the internal structure of the heart, which finally diagnosed STC.

LVNC, also known as hypertrabeculated left ventricular myocardium, is the reticular thick trabecular hyperplasia on the membranous surface of the left ventricle arising when the development of endocardium and cardiomyocytes stops in the early embryonic period. The recessed crypts form between the trabeculae communicate with the ventricle. LVNC is sporadic or familial, and has genetic mutations involving multiple sites. LVNC can manifest as isolated left ventricular LVNC or coexist with other congenital heart diseases. The clinical manifestations are mainly heart failure, arrhythmia, and heart cavity internal thrombosis and embolic events in the arterial system [14].

Distinguished from LVNC, CMR of STC usually shows normal or slightly elevated T1 (longitudinal relaxation time), no myocardial edema in T2 (transverse relaxation time) mapping, and no late-stage gadolinium enhancement evidence of myocardial fibrosis or previous infarction. Therefore, it is mostly believed that STC can be characterized as a “cardiomyopathy” to emphasize the compactness and special protrusion structure of the left ventricular muscle bridge, simulating the saw-tooth like projection [15]. However, few reports provide cardiac biopsy or gross specimens. Therefore, the internal myocardial arrangement and developmental form of STC still require additional study.

## 5. Histological and Genetic Evaluation of Saw-Tooth Cardiomyopathy

By application of genomics, Julie Proukhnitzky *et al*. [10] sequenced 71 cardiomyopathy genes in an STC patient, including titin, exons, and flanking regions, but American College of Medical Genetics (ACMG) criteria matched no related pathogenic gene variants. In the application of pathology, Felix K. Wegner *et al*. [11] performed a myocardial biopsy on a male patient with STC combined with complete left bundle branch block, and found no further obvious abnormality. The patient remained clinically stable during 52 months of follow-up. Davlouros’ [2] first report on a 2-month-old baby with heart failure indicated that STC may have a strong background of congenital genetic abnormalities. However, no myocardial saw-tooth like changes have yet been found in first-degree relatives of any known STC case.

We summarize all published reports in Table 1. Male patients with STC accounted for the majority (80.0%), and the average age at diagnosis was 35.8 years old. Note that these two traits were similar to LVNC: in 2018, Shijie Li *et al*. [16] surveyed a gene sequencing cohort of 100 LVNC patients and found that males accounted for 72%, and the average age of diagnosis was 39.5 years. Shijie Li *et al*. [16] documented a total of 42 pathogenic variants in 38 patients. After a median 4.2-year follow-up, they found that cardiomyopathy gene mutations (*TTN*,* MYH7*, *MYBPC3*, *DSP*, etc.) were significantly related to the risk of death or heart transplantation (hazard ratio = 2.49; 95% confidence interval 1.15–5.37, *p *= 0.020) in LVNC. Accordingly, clinicians should construct detailed long-term follow-up schemes to reduce future risk of cardiogenic events in STC patients.

There are reasons to suspect that in the detection of genetic sequencing and pathological results of STC patients, there may be a certain selection offset due to the progression of different heart failure conditions, resulting in false negatives in sequencing results or pathological tests, influencing the finding that the current incidence of STC is rare and the number of patients tested is too small. Therefore, the detection range of cardiomyopathy genes should be properly expanded in such patients, not only limited to LVNC, or other known abnormally differentiated cardiomyopathy related genes, such as dilated cardiomyopathy, hypertrophic cardiomyopathy, amyloid cardiomyopathy and others. Using a full penetrative whole-exome sequencing (WES) design scheme to look for unrevealed mutation gene loci, and expansion of the tested sample size in the future could provide a broader research prospect for the diagnosis and pathogenesis of STC.

## 6. Complications and Prognosis of Saw-Tooth Cardiomyopathy

Left ventricular insufficiency is the most common complication and admission diagnosis among the STC population (80%), followed by left bundle branch block (40%), stroke/TIA/patent foramen ovale (30%), and mitral valve prolapse (10%). When heart failure diagnosis is clear, symptomatic treatment of ventricular insufficiency can achieve obvious benefits. By receiving regular outpatient follow-ups with a scheduled medicine treatment plan, patients can have recovery of cardiac function and improvement of clinical symptoms [2, 10].

The saw-tooth like changes in the endocardial structure may have a certain impact on the intracardiac conduction system, which manifested as delayed bundle branch activation in four patients. Cardiac resynchronization therapy (CRT), a cardiac pacing method used for ventricular activation [17], showed good effect in the reported 34-year-old female STC patient who had left ventricular systolic synchronized dysfunction [12]. STC involved with atrioventricular septal defect may be an abnormal shunt channel that can cause cerebrovascular accidents. Similar to the current LVNC research regarding complications, including atrial fibrillation, severe heart failure, previous embolism event or current intracardiac thrombosis, anticoagulation therapy may be necessary [18]. Under other circumstances, Halioui M *et al*. [9] proposed in a 16-year-old STC girl involving the left ventricular myocardium at the bottom of the valve that it was complicated with a mitral valve prolapse, as the posterior papillary muscles constantly hitting the protruding endocardial surface can aggravate mitral valve regurgitation. Therefore, mitral valve repair surgery tailored to the particular patient may be an appropriate choice.

These results indicate that STC is usually accompanied by complications. At first diagnosis, attention should be paid to distinguish STC from LVNC. To date there is no direct evidence to determine whether STC is a new cardiomyopathy different from LVNC or is a special differentiated subtype of LVNC. More clinical reports, histological identification and WES can further evaluate or even improve the detection and diagnosis of STC and continue modifying the understanding of this disease. CMR is a foremost diagnostic tool in judging the ventricular structure and function, which can navigate the management of STC in heart failure and cardiogenic complications, clarifying the prognosis of patients.

## 7. Conclusions

Saw-tooth cardiomyopathy is a rare left ventricular cardiomyopathy characterized by multiple projections originating from the interventricular septum, tethering to the ventricular wall, giving the heart a “saw-tooth” appearance on imaging. We summarize the clinical evidence from the current decade, which may enhance the clinician’s detection and diagnosis of this disease in the future.
